# 

**Published:** 1878-09

**Authors:** 


					﻿SEPTEMBEB, 1878.
TWENTY-FIFTH YEAR—No 297.
IIAT.L’S
Journal of Health.
PUBLISHED AT 74 FOURTH AVENUE,
E. H. GIBBS, A.M., M.D., Editor.
Medical Office—139 East Eighth Street, Near Broadway.
OFFICE HOURS—FROM 10 A.M. TO 3 P.M.
AGE NTS FOR THE TRADE J
AMERICAN NEWS COMPANY AND NEW YORK NEWS COMPANY.
Terms, $1.50 a Year, or $1.00 for Eight Months. Postage paid.
SINGLE NUMBER, 15 CENTS.
				

